# Adiponectin–leptin ratio for the early detection of lean non-alcoholic fatty liver disease independent of insulin resistance

**DOI:** 10.1080/07853890.2023.2179106

**Published:** 2023-02-15

**Authors:** Chia-Wen Lu, Kuen-Cheh Yang, Yu-Chiao Chi, Tsan-Yu Wu, Chien-Hsieh Chiang, Hao-Hsiang Chang, Kuo-Chin Huang, Wei-Shiung Yang

**Affiliations:** a Graduate Institute of Clinical Medicine, College of Medicine, National Taiwan University, Taipei, Taiwan; b Department of Family Medicine, National Taiwan University Hospital, Taipei, Taiwan; c Department of Family Medicine, College of Medicine, National Taiwan University, Taipei, Taiwan; d Department of Internal Medicine, National Taiwan University Hospital, Taipei, Taiwan; e Department of Family Medicine, National Taiwan University Hospital, Hsin-Chu, Taiwan

**Keywords:** Lean NAFLD, leptin, adiponectin, adiponectin-leptin ratio, insulin resistance

## Abstract

**Background:**

Lean Non-alcoholic Fatty Liver Disease (NAFLD) shares a similar disease burden to those of their overweight counterparts and should be detected early. We hypothesized that the adiponectin–leptin ratio (AL ratio) could be a good marker for early detection of lean NAFLD independent of insulin resistance.

**Materials and methods:**

A total of 575 adults without diabetes were enrolled in a community-based study. The subjects were stratified into the lean controls, lean NAFLD, simple overweight/obesity and overweight/obesity NAFLD groups according to body mass index (BMI) and ultrasonographic fatty liver indicators. Serum adiponectin and leptin levels were measured by enzyme-linked immunosorbent assay. Multivariate logistic regression analyses were performed to estimate the odds ratio of having NAFLD in relation to the tertiles of serum AL concentration after adjustment. Receiver operating characteristic (ROC) analyses were applied to evaluate the diagnostic performance of the AL ratio for NAFLD.

**Results:**

The mean age of the participants was 42.8 ± 11.5 years. Comparing with the lean controls, the odds of having lean NAFLD for the highest versus the lowest tertile of AL ratio was 0.28(95%CI: 0.12–0.69) after adjustment. Putting AL ratio, BMI, triglyceride, AST/ALT ratio to the diagnosis performance of NAFLD, the ROC was 0.85 (95% CI: 0.82–0.88), 0.83 (95% CI 0.78–0.87) and 0.86 (95% CI 081–0.91) for all NAFLD, NAFLD in women and NAFLD in men, respectively. (*p* < .001).

**Conclusions:**

The study revealed that the AL ratio could be a good biomarker to early distinguish lean NAFLD patients from lean controls independent of insulin resistance. [AQ3]

**Key messages**
1. The prevalence of non-alcoholic fatty liver disease (NAFLD) increases globally and is related to liver diseases and metabolic dysfunctions. Lean subset of NAFLD shares a similar disease burden to those of their overweight counterparts and should be detected early.
2. Adiponectin–leptin ratio were associated with the severity of steatosis and was a predictor of obese NAFLD better than each single adipokine. To date, there is no investigation that explores specifically for the relationship between lean NAFLD and AL ratio.
3. Our study found that adiponectin–leptin ratio is a sole independent marker regardless of insulin resistance in lean NAFLD. Having lean NAFLD for the highest versus the lowest tertile of adiponectin–leptin ratio was 0.28(95%CI: 0.12–0.69) after adjustment of age, sex, current smoking, exercise habits, HOMA-IR and AST/ALT. ROC for the NAFLD performance is good for the early detection (0.85; 95% CI: 0.82–0.88). Further rigorous investigation is necessary and should be promptly performed.

## Introduction

The prevalence of nonalcoholic fatty liver disease (NAFLD), recently termed metabolic dysfunction-associated fatty liver disease (MAFLD), is increasing globally [1]. The incidence of NAFLD is estimated to be 28–52 per 1,000 person/years, and the prevalence is approximately a quarter of adult population [2]. NAFLD is a formidable public health issue that is widely associated with hepatic and extrahepatic comorbidities or complications, such as cirrhosis, hepatocellular carcinoma, diabetes, metabolic syndrome and cardiovascular diseases [3]. Moreover, the core pathophysiology of insulin resistance and increased adiposity of NAFLD attribute its cause to metabolic dysregulation with significant liver involvement [4]. As we know, the metabolic phenotype of NAFLD is characterized mainly by insulin resistance due to the hepatic oversupply with sugar, lipid and etc, while the genetic component is characterized by the impaired hepatic mitochondrial function, leading to chronic inflammation [5]. The pathogenesis of NAFLD represents the metabolic dysfunction clinically of a complex interplay between lifestyle, environmental and genetic factors along with a key role for epigenetic changes [6]. As a fatty live disease mainly composed of metabolic dysfunctions, the prevalence of NAFLD is approximately one quarter in the world in which composed of 50% of T2D, approximately 70% among cardiovascular diseases and more than 90% of severely obese patients [7]. Therefore, an endorsement by more than 1000 specialists over 134 countries have emphasized that MAFLD is an overarching term associated with metabolic dysregulation [8].

Paradoxically, there has been a growing subset of patients who are inflicted with NAFLD, but their body mass index (BMI) is classified as lean (defined as BMI <25 in the Western region and BMI <23 in the Asian region) [9]. Lean NAFLD varies in prevalence among different ethnic groups or with diagnostic approaches, accounting for 5% to 8% in Caucasian subjects and 16% to 18% in the Asia-Pacific region [10]. Without obesity as a prerequisite for NAFLD, lean NAFLD shares similar severities of advanced diseases and mortality similar to its obese counterpart [11]. Therefore, the new definition of MAFLD, i.e. NAFLD required an evidence of hepatic steatosis, detected either by imaging techniques, blood biomarkers/scores or by liver histology and involves one of the three following phenotypes, (1) overweight/obesity, (2) the presence of type 2 diabetes mellitus or (3) lean subjects with evidence of metabolic dysregulation [12]. Since liver biopsy cannot be applied widely, ultrasound is the most practical imaging modality for screening NAFLD [13]. Nevertheless, a reliable biomarker or score is urgently needed for early detection and diagnosis of NAFLD, especially for easily ignored populations, i.e. lean NAFLD. Currently, the fatty liver index (FLI), which incorporates BMI, waist circumference (WC), gamma-glutamyl transferase and triglyceride levels, may be the most established index for scoring NAFLD [14]. The plasma cytokeratin 18 (CK18) fragment level is the most extensively evaluated biomarker of steatohepatitis and is a marker of hepatocyte apoptosis [15]. However, none of the above biomarkers/scores are specific for early detection of lean NAFLD.

Adiponectin and leptin were discovered in the 1990s. Since then adipose tissue has gradually transformed from a simple energy reservoir to a highly active endocrine organ [16,17]. Leptin is positively correlated with obesity and insulin resistance [18], while adiponectin shows a good ability to enhance insulin sensitivity and counteract the development of diabetes [19,20]. Additionally, leptin dually exerts antisteatotic proinflammatory and profibrogenic actions for NAFLD. The net effect however remains unclear [21]. In contrast, adiponectin consistently promotes anti-inflammatory and antifibrotic activity [22]. Consequently, adiponectin to leptin ratio was assumed to correlate negatively with low-grade chronic inflammation [23], atherosclerosis risk [24] and cardiovascular disorders [25,26]. A few human studies have elaborated the association between the adiponectin, leptin or AL ratio and NAFLD [27,28] while few were related to lean NAFLD. No matter obese or not, adiponectin is a biomarker for NAFLD subjects indicating the progression to steatohepatitis in a biopsy proven study [29] and the development of NAFLD in a Korea cohort [30]. And, lean subjects with evidence of NAFLD have lower adiponectin concentrations than lean controls in Caucasian populations [6]. In the other hand, leptin levels reflect total body fat and insulin resistance [31] that correlate positively with hepatic steatosis in diabetes subjects [32]. Taking together, AL ratio were associated with the severity of steatosis in a Japanese study [27] and was a predictor of NAFLD in obese adults that correlated with liver function and insulin resistance better than each single adipokine [28]. To date, there is no investigation that explores specifically for the relationship between lean NAFLD and AL ratio.

Theoretically, lean subjects with normal BMIs and adiposity should have higher circulating adiponectin and lower circulating leptin. However, NAFLD itself is a chronic process of liver inflammation which may alter the level of circulating adiponectin and leptin. As the result, whether the AL ratio is the same for lean subjects with or without NAFLD remains unclear. We hypothesized that the AL ratio could distinguish patients with lean NAFLD from those without NAFLD in the very early stage independent of insulin sensitivity. Therefore, we conducted this community-based study to enrol young adults without diabetes and applied strict ultrasound scoring to investigate the relationship between AL ratio in the four groups: lean controls, lean NAFLD, simple overweight/obesity and overweight/obesity NAFLD groups. We also applied ROC analyses to find a most suitable diagnostic performance of NAFLD using AL ratio and available biomarkers in clinical setting.

## Materials and methods

### Study subjects

This study was conducted cross-sectionally in a community in Northern Taiwan. All the participants enrolled when they received a regular health check-up in National Taiwan University Hospital, Hsin-Chu branch. All the subjects completed standardized questionnaires through individual interview regarding socio-demographics, smoking, drinking, exercise and medical history. Subjects who had a history of diabetes, were taking antihyperglycemic agents or insulin or fasting serum glucose ≥126 mg/dl or haemoglobin A1c ≥6.5% were excluded. In total, 575 adults older than 20 years were enrolled. Weight, height and Blood pressure (BP) were measured by calibrated, electronic stadiometers and sphygmomanometers. WC was measured horizontally through the middle point between the upper border of iliac bones and the lower border of the ribs. Body fat percentage was measured through bioelectrical impedance analysis by a portable body composition analyser (TANITA BC-418, Japan). Abdominal ultrasonography was performed by three experienced physicians using a 3.5–5 MHz transducer and a high-resolution B-mode scanner (Hitachi Aloka ProSound Alpha 6, Japan). The severity of NAFLD was calculated using the US-FLI score [9]. The details about the including and excluding criteria, questionnaires, the scoring of fatty liver by abdominal ultrasonography and blood analyses please refer to our published study [33]. Informed consent forms were signed. This study was approved by the Institutional Review Board of National Taiwan University Hospital (IRB NO. 201210012RIC).

### Definition of lean and NAFLD groups

The cut-off points for BMI categories in Taiwan are defined as follows: <18.5 kg/m^2^: underweight, 18.5–23.9 kg/m^2^: normal weight, 24–26.9 kg/m^2^: overweight, ≥27 kg/m^2^: obesity [34]. The subjects were then divided into the following groups [1]: lean controls: US-FLI score <2, BMI < 24 kg/m^2^ [2]; lean NAFLD group: US-FLI score ≥2, BMI< 24 kg/m^2^ [3]; simple overweight/obesity group: US-FLI score <2, BMI ≥24 kg/m^2^; and [4] overweight/obesity NAFLD group: US-FLI score ≥2, BMI ≥24 kg/m^2^ [33].

### Blood analysis

Serum adiponectin (As One International INC, Santa Clara, CA, USA) was diluted to 10x during pre-treatment, incubated at 100 °C for 5 min and then diluted to 5100x finally. Serum leptin (R&D Inc. Minneapolis, USA) was diluted to 30x using dilution buffer. The limit of detection (LOD) was 23.4 pg/mL and 7.8 pg/mL for adiponectin and leptin, respectively. The intra-assay and inter-assay coefficients of variation (CVs) were all less than 5%. Both adiponectin and leptin were then measured by enzyme-linked immunosorbent assay following manufacturer’s protocol as previously described [35].

### Statistical analysis

Data are presented as the mean ± SD for continuous variables and number (percentage) for categorical variables. Differences between the four groups were examined using the chi-squared test for categorical variables and one-way analysis of variance (ANOVA) for continuous variables. Tukey’s post hoc analysis was applied to examine the differences among the healthy control, lean NAFLD, overweight controls and overweight NAFLD groups in terms of basic demographic characteristics, leptin, adiponectin and AL ratio. Multivariate linear regression analyses were performed to estimate the relationship between the AL ratio and metabolic factors. We put lean controls, lean NAFLD, simple overweight/obesity and overweight/obesity NAFLD groups as dependent variables and the tertiles of AL ratio as an independent variable. Then, multivariate logistic regression models were applied to examine the odds of having NAFLD in relation to the tertiles of AL ratio after adjustments age, sex, current smoking, exercise habits, HOMA-IR and AST/ALT ratio. We performed receiver operating characteristic (ROC) analysis with the area under the ROC curve (AUC) to evaluate the diagnostic performance of the AL ratio for NAFLD. All analyses were performed using SPSS statistical software (V.17, SPSS, Chicago, Illinois, USA). A *p* value of <.05 was considered to be statistically significant.

## Results

The basic characteristics of the participants are shown in Table 1. The mean age of the participants was 42.8 ± 11.5 years, and 61.3% of the participants were female. Of the 575 subjects included, 200 subjects (34.8%) had overweight/obesity NAFLD, and 105 subjects (18.3%) had lean NAFLD. Since we excluded diabetes, our study group had a high proportion of metabolically healthy subjects (MetS factors: 1.08 ± 1.11). Tukey’s post hoc analysis was performed to test the differences between groups. Importantly, the AL ratio can specifically tell the lean control from the lean or overweight/obesity NAFLD group rather than adiponectin or leptin alone. To compare the lean NAFLD group and simple overweight/obesity group, we found that their BMI, fat percentage and waist circumference were significant differences while there were no differences in any metabolic parameters, including blood pressure, lipid profile, glucose, insulin resistance or inflammatory biomarkers such as AST, ALT and CRP.

**Table 1.** Baseline characteristics among the lean controls, lean NAFLD, simple overweight/obesity and overweight/obesity NAFLD groups.

	BMI < 24		BMI ≥ 24	
	Lean controls	Lean NAFLD	Simple overweight/obesity	Overweight/obesity NAFLD
	*N* = 217	*N* = 105	*N* = 53	*N* = 200
Age (years)	41.20 ± 10.94	42.96 ± 11.59	44.38 ± 11.34	43.33 ± 11.80
Male (%)	45(20.7)^c,d^	36(34.3) ^d^	25(47.2) ^a^	113(56.5) ^a, b^
Smoke (%)	14(6.5)^d^	11(10.5)	5(9.4)	33(16.5) ^a,^
Exercise (%)	95(43.8)	45(42.9)	26(49.1)	86(43.0)
BMI (kg/m^2^)	20.66 ± 1.80 ^b, c, d^	21.83 ± 1.54 ^a, c, d^	25.95 ± 1.75 ^a, b, d^	27.98 ± 3.91 ^a, b, c^
Fat percentage (%)	27.54 ± 8.08 ^b, c, d^	30.76 ± 7.33 ^a, d^	33.89 ± 7.12 ^a^	36.15 ± 7.89 ^a, b^
WC (cm)	73.13 ± 6.13 ^b, c, d^	77.56 ± 6.58 ^a, c, d^	85.72 ± 5.92 ^a, b, d^	90.40 ± 7.88 ^a, b, c^
Systolic BP	115.80 ± 15.52 ^b, c, d^	122.12 ± 15.15 ^a, d^	122.63 ± 17.16 ^a, d^	129.99 ± 15.12 ^a, b, c^
Diastolic BP	73.00 ± 11.18 ^b, c, d^	77.50 ± 9.46 ^a, d^	78.06 ± 13.91 ^a^	81.75 ± 11.93 ^a, b^
TCHO (mg/dL)	189.78 ± 33.99 ^d^	197.60 ± 40.05	194.00 ± 29.23	202.64 ± 35.34 ^a^
TG (mg/dL)	74.00 ± 35.43 ^b, d^	109.36 ± 79.36 ^a, d^	96.02 ± 43.21 ^d^	157.51 ± 113.57 ^a, b, c^
HDL-C (mg/dL)	66.78 ± 14.96 ^b, c, d^	57.13 ± 13.35 ^a, d^	59.09 ± 13.23 ^a, d^	49.93 ± 12.61 ^a, b, c^
LDL-C (mg/dL)	114.12 ± 31.13^b, d^	126.20 ± 37.28 ^a^	122.49 ± 29.21	132.86 ± 32.16 ^a^
Glucose (mg/dL)	82.65 ± 8.66 ^d^	85.50 ± 8.65 ^d^	86.51 ± 9.83 ^d^	89.39 ± 9.43 ^a, b, c^
Insulin (μIU/mL)	5.13 ± 3.28 ^d^	6.71 ± 5.24 ^d^	7.15 ± 3.87 ^d^	11.30 ± 9.03 ^a, b, c^
HOMA-IR	0.66 ± 0.42 ^d^	0.86 ± 0.65 ^d^	0.92 ± 0.49 ^d^	1.44 ± 1.11 ^a, b, c^
GOT (U/L)	20.46 ± 6.86 ^d^	21.75 ± 7.03 ^d^	21.57 ± 6.00 ^d^	25.40 ± 10.07 ^a, b, c^
GPT (U/L)	17.22 ± 9.48 ^b, d^	23.96 ± 16.61 ^a, d^	12.48 ± 10.74 ^d^	35.69 ± 27.84 ^a, b, c^
CRP (mg/dL)	0.114 ± 0.320 ^d^	0.091 ± 0.123 ^d^	0.173 ± 0.277	0.207 ± 0.250 ^a, b^
Metabolic factors (n)	0.39 ± 0.62 ^b, c, d^	0.92 ± 0.90 ^a, d^ -	1.15 ± 0.91 ^a, d^	2.01 ± 1.24 ^a, b, c^
MetS (%)	1(0.5) ^d^	6(6.2) ^d^	4(8.5) ^d^	56(28.0) ^a, b, c^
Adiponectin(μg/mL)	18.13 ± 8.55 ^b, d^	13.60 ± 8.00 ^a, d^	15.01 ± 8.82 ^d^	9.82 ± 5.71 ^a, b, c^
Leptin (ng/mL)	8.16 ± 6.30 ^c, d^	9.42 ± 7.21 ^d^	12.48 ± 10.74 ^a^	15.20 ± 11.35 ^a, b^
AL ratio (x10^3^)	6.43 ± 18.36 ^b. d^	2.26 ± 1.93 ^a^	2.23 ± 2.32	1.13 ± 1.14 ^a^

Abbreviations: NAFLD: non-alcoholic fatty liver disease; BMI: body mass index; WC: waist circumference; BP: blood pressure; TCHO: total cholesterol; TG: triglycerides; HDL-C: high-density lipoprotein cholesterol; LDL-C: low-density lipoprotein cholesterol; HOMA-IR: homeostasis model assessment of insulin resistance; GOT: glutamic oxaloacetic transaminase; GPT: glutamic pyruvic transaminase; CRP: C-reactive protein; MetS: metabolic syndrome; AL ratio: adiponectin-leptin ratio.

*Notes:* Data are presented as the mean ± SD for continuous variables and number (percentage) for categorical variables. Differences between the four groups were examined using the chi-squared test for categorical variables and one-way analysis of variance (ANOVA) for continuous variables. Tukey’s post hoc analysis was applied to examine the differences among the healthy control, lean NAFLD, overweight controls and overweight NAFLD groups in terms of basic demographic characteristics, leptin, adiponectin and AL ratio.

The four groups were represented with ^a^: lean controls; ^b^: lean NAFLD; ^c^: simple overweight/obesity; ^d^: overweight/obesity NAFLD. If a significant level *p* < .05 was achieved between any two of the four groups, a superscript was added to the corresponded columns.

To further clarify the association between each factor of metabolic syndrome and the AL ratio, we applied multivariate linear regression models after adjusting for age and sex (Table 2). The AL ratio was negatively associated with body fat percentage, BMI, WC, SBP, DBP, TG, glucose and HOMA-IR (all *p* < .001) while positively associated with HDL (*p* < .001). These results impressed that AL ratio is a consistent and strong biomarker for detecting metabolic dysfunction.

**Table 2.** Relation between the serum adiponectin-leptin ratio and metabolic factors in multivariate linear regression models after adjusting for age and sex.

Variables	Model 1	Model 2	Model 3	Model 4	Model 5	Model 6	Model 7	Model 8	Model 9
	ß (SE) *p* Value	ß (SE) *p* Value	ß (SE) *p* Value	ß (SE) *p* Value	ß (SE) *p* Value	ß (SE) *p* Value	ß (SE) *p* Value	ß (SE) *p* Value	ß (SE) *p* Value
BMI (kg/m^2^)	−0.252(0.108) <.001								
Fat (%)		−0.365(0.060) <.001							
WC (cm)			−0.296(0.050) <.001						
SBP (mmHg)				−0.141(0.030) .001					
DBP (mmHg)					−0.133(0.040) .002				
HDL-C (mg/dl)						0.158(0.033) <.001			
TG (mg/dl)							−0.165(0.005) <.001		
Glucose (mg/dl)								−0.102(0.028) .015	
HOMA-IR									−0.203(0.260) <.001

Abbreviations: NAFLD: non-alcoholic fatty liver disease; BMI: body mass index; WC: waist circumference; SBP: systolic blood pressure; DBP: diastolic blood pressure; TG: triglycerides; HDL-C: high-density lipoprotein cholesterol; LDL-C:low-density lipoprotein cholesterol; HOMA-IR: homeostasis model assessment of insulin resistance.

Knowing that the AL ratio was a good parameter in relation to each factor of metabolic syndrome, multivariate logistic regression models were performed to explore the odds of having NAFLD in relation to the tertiles of serum AL ratio (Table 3). The OR of having NAFLD for the highest versus the lowest tertile of AL ratio was 0.34 (95% CI: 0.17–0.71; *p* for trend < .001). After further adjustment of AST/ALT ratio, the OR of having NAFLD for the highest versus the lowest tertile of AL ratio was 0.37 (95% CI: 0.18–0.77, *p* for trend .008).

**Table 3.** Odds ratios of having NAFLD in relation to the serum tertile of adiponectin-leptin (AL × 10^3^) ratio using multiple logistic regression analyses.

	AL × 10^3^ ratio <0.91 *N* = 190	0.91≤ AL ×10^3^ ratio <2.36 *N* = 193	AL × 10^3^ ratio ≥2.36 *N* = 192	*p* for trend
Model 1	1.00	0.28(0.17–0.44) **	0.07(0.04–0.12) **	<.001
Model 2	1.00	0.58(0.34–1.00) *	0.22(0.12–0.43) **	<.001
Model 3	1.00	0.66(0.37–1.12)	0.34(0.17–0.71) **	<.001
Model 4	1.00	0.67(0.38–1.21)	0.37(0.18–0.77) *	.008

*Notes:* Model 1: adjusted for age, sex, current smoking and exercise habits; Model 2: adjusted for variables in Model 1, plus BMI; Model 3: adjusted for variables in Model 2, plus HOMA_IR; Model 4: adjusted for variables in Model 2, plus GOT/GPT ratio.

* *p* < .05; ** *p* < .001.

Stratified by BMI, the ORs of having NAFLD derived from multiple logistic regression analyses in tertiles of serum AL ratio are shown in Table 4. When BMI <24 kg/m^2^, the OR of having NAFLD for the highest versus the lowest tertile of AL ratio after adjustment was 0.28 (95% CI: 0.12–0.69; *p* for trend .005). When BMI ≥24 kg/m^2^, the OR of having NAFLD for the highest versus the lowest tertile of AL ratio after adjustment was 0.30 (95% CI: 0.09–0.96; *p* for trend .043).

**Table 4.** Odds ratios of having NAFLD in relation to serum tertile of adiponectin leptin level using multiple logistic regression analyses, stratification by BMI.

Lean NAFLD				
	AL <0.91 *N* = 53	0.91 ≤ AL <2.36 *N* = 120	AL ≥2.36 *N* = 149	*p* for trend
Model 1	1.00	0.58(0.29–1.14)	0.16(0.08–0.36) **	<.001
Model 2	1.00	0.59(0.29–1.21)	0.26(0.11–0.61) *	.002
Model 3	1.00	0.62(0.30–1.29)	0.28(0.12–0.69) *	.005

Overweight NAFLD				
	AL <0.91 *N* = 137	0.91 ≤ AL <2.36 *N* = 73	AL ≥2.36 *N* = 43	*p* for trend
Model 1	1.00	0.34(0.14–0.80) *	0.11(0.04–0.30) **	<.001
Model 2	1.00	0.59(0.23–1.51)	0.28(0.09–0.89) *	.031
Model 3	1.00	0.61(0.23–1.58)	0.30(0.09–0.96) *	.043

*Notes:* Model 1: adjusted for age, sex, current smoking and exercise habits; Model 2: adjusted for variables in Model 2, plus HOMA_IR; Model 3: adjusted for variables in Model 2, plus GOT/GPT ratio.

*p < .05; **p < .001.

**Figure 1.**
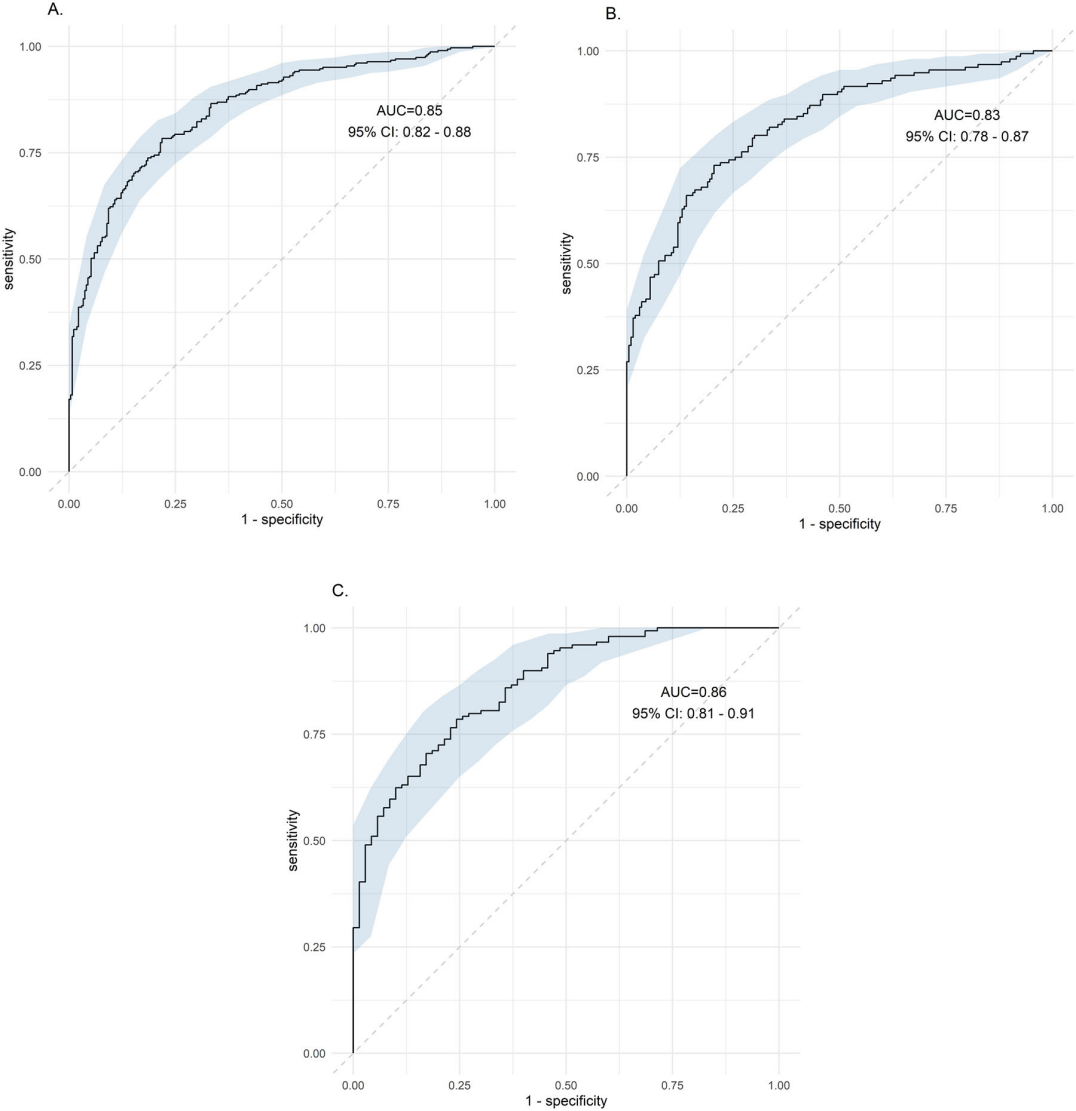
Receiver operating characteristic (ROC) for the diagnosis of NAFLD. Except adiponectin-leptin ratio, BMI, triglyceride and AST/ALT ratio were selected. (A) All subjects, AUROC was 0.85 (95% CI: 0.82–0.88), (B) female subjects, AUROC was 0.83 (0.78–0.87), and (C) male subjects, AUROC was 0.86 (081–0.91). All *p* < .001.

The AL ratio, BMI, triglyceride and AST/ALT ratio were selected for the diagnosis performance of NAFLD using ROC analysis curve. For all subjects, the AUROC was 0.85 (95% CI: 0.82–0.88). For female and male, AUROC was 0.83 (0.78–0.87) and 0.86 (081–0.91), respectively (all *p* < .001) (Figure 1).

## Discussion

A logical inference between lean NAFLD and AL ratio was well demonstrated in the study. First, we demonstrated that in this population that was younger and healthier, the AL ratio was indeed a strong and good parameter in relation to each metabolic factor and HOMA-IR. Then, the association between the serum AL ratio and the risk of NAFLD was examined. In the section of crude OR, both the lean NAFLD and overweight/obesity NAFLD groups showed a decreased risk from the lowest tertile of AL ratio to the highest tertile of AL ratios compared with that of the lean controls and simple overweight/obesity groups, respectively (*p* for a trend <.001). Then, we removed the effects of HOMA-IR and AST/ALT ratio to determine the amount of residual effect differences that were left between the case and controls (lean controls vs. lean NAFLD; simple overweight/obesity vs. overweight/obesity NAFLD). As a result, a persistent lower risk of NAFLD was found in the lowest tertile of AL ratio to the highest tertile of AL ratios (*p* for trend <.05). The AUROC curve also performed very well at the level of 0.83–0.86.

Adipose tissue, as a sophisticated endocrine organ, performs crosstalk with the liver by circulating adipokines for the development of NAFLD [36,37]. Among adipokines, adiponectin and leptin have contrary roles in relation to BMI. As the gradual transition from lean to overweight to obesity occurred, which is associated with the accumulation of adiposity, the serum adiponectin level decreased in parallel with the increase in serum leptin levels [25,38]. In addition to altering insulin sensitivity and the function of adipocyte lipid storage, adiponectin and leptin are related to inflammatory or anti-inflammatory functions [20,39]. Some observational studies have demonstrated that the linkage between anti-inflammation and adiponectin is at least partially independent of obesity [40], and this result is consistent with our study. Consequently, the AL ratio has been suggested to be a marker of low-grade chronic inflammation in populations with impaired insulin functions and obesity [25,27,41]. Some studies propose that the AL ratio is positively associated with arteriosclerosis, intima media thickness of the common artery and CVD [26,42]. A Japanese health survey delineated cross-sectionally that the AL ratio was associated with the severity of steatosis by ultrasonography [27]. Another study elucidated that the AL ratio could be a noninvasive predictor of NAFLD in obese children, which better correlates with weight and HOMA-IR than each single adipokine [28]. Compared to MALFD, leptin is more robust in the effect of obesity, while adiponectin could interfere with the presentation of NAFLD regardless of HOMA-IR and adiposity. Therefore, the AL ratio could be independently used to distinguish the lean NAFLD individuals from the lean control individuals.

Since 2020, MAFLD has been used as the main terminology instead of NAFLD [8,43]. It has been indicated that although lean NAFLD patients are younger and have fewer metabolic clinical features, they share similar histological severity, comorbidities and mortality with NAFLD patients [44]. Because lean NAFLD subjects develop fatty liver disease prior to becoming overweight or having increased adiposity, we could utilize image modality or biomarker rather than BMI for early detection. We excluded diabetes because its pathophysiology could be another pathway and progression trajectory [8,45]. We enrolled early-stage NAFLD patients with less metabolic syndromes. Since liver fat accumulation and chronic inflammation are very sensitive and early indicators in these subsets, the AL ratio was suggested to be a good early classifier for lean NAFLD.

Lean NAFLD is more prevalent in Asia area that reflects ethnic differences and genetic variants [46]. In a recent meta-analysis, the prevalence of lean NAFLD among non-obese population was up to 40.75% in Asian [47]. In line with our study, we found that 105 of the 322 lean subjects (33%) had lean NAFLD in our population. And, the prevalence of lean NAFLD in the NAFLD subjects in Asia is varied, ranging from 12% to 47% which was also consistent with our finding (105 of 305, 34.4%) [48].

NAFLD composed of 50% of T2D [7] and encountered a changing of terminology to MAFLD after 2021(8). MAFLD separated diabetes as a unique category from the other two categories, obese or lean with metabolic dysfunction, for its different pathophysiology [12]. Compared with our previous published article extracted from the same population [33], we excluded diabetes in this study for better understanding and detecting the lean NAFLD. General speaking, the metabolic phenotype of NAFLD is characterized mainly by insulin resistance while the genetic component is characterized by the impaired hepatic mitochondrial function [5]. That’s why we chose adiponectin and leptin, both as adipokines and hepatokines, to detect lean NAFLD. Furthermore, we performed an AUROC analysis to consolidate the hypothesis that adiponectin/leptin ratio is good performance in NAFLD detection.

There are some limitations in our study. First, we did not perform liver biopsy. Although liver biopsy is the gold standard for NAFLD, the high prevalence and variable presentation of NAFLD make performing biopsies less practical. Nevertheless, we applied a strict echo score, the US-FLI, which has been well validated and applied in previous studies. Although the ultrasonographic approach cannot determine the severity of NAFLD, it has been validated by US-FLI as a reliable dichotomous screening tool for NAFLD [38]. In addition, US-FLI has been applied extensively as a substitute modality for the diagnosis of NAFLD in the real world [9]. Second, this was a cross-sectional study, and we could only determine the association rather than a causal relationship between the AL ratio and NAFLD. We tried to enrol early NAFLD patients, but we did not record the duration of NAFLD that may potentially influence the serum AL ratio. We adjusted insulin resistance as a pivotal step to demonstrate that persistent low-grade inflammation of lean NAFLD plays a key role and could independently be related to the AL ratio; however, we applied an indirect measurement by the equation that was transformed by fasting glucose and insulin instead of a standard glucose clamp technique. Although we demonstrated a significant association between AL ratio and NAFLD, focussing on lean NAFLD, the cross-sectional study could not infer an early detection. For a better detection model or further validation model, a longitudinal cohort is warranted.

## Conclusions

In conclusion, this was the first investigation to link the negative association between serum AL ratio and lean NAFLD. Our study found that the AL ratio is a sole independent marker regardless of insulin resistance in lean NAFLD. Combination of AL ratio, BMI as well as triglyceride and AST/ALT ratio, ROC for the NAFLD performance is good for the early detection. Further rigorous investigation is necessary and should be promptly performed.

## Data Availability

The datasets used and/or analysed during the current study are available from the corresponding author on reasonable request.
